# Isolation and complete genome sequence of *Aeromonas* bacteriophage Gekk3-15

**DOI:** 10.12688/f1000research.144007.1

**Published:** 2024-04-24

**Authors:** A.K. Golomidova, E.E. Kulikov, A.S. Kuznetsov, P. Yu. Pechenov, I.S. Belalov, A.V. Letarov, E.E. Galyov

**Affiliations:** 1 Winogradsky Institute of Microbiology, Research Center “Fundamentals of Biotechnology” RAS, Moscow, Russian Federation; 2 Genetics and Genome Biology, University of Leicester, Leicester, UK

**Keywords:** bacteriophage, Aeromonas veronii, aquaculture, phage therapy

## Abstract

Bacteria of the genus *Aeromonas*, especially *A. hydrophila* and *A. veronii* are recognized as important fish pathogens that cause significant economic losses in aquaculture. Environmentally friendly bacteriophage-based solutions for the treatment of fish and for the reduction of colonization by pathogenic bacteria in production facilities are currently in high demand. The bacteriophage Gekk3-15 was isolated during a search for novel phage strains potentially suitable for *Aeromonas* biocontrol applications. Genome sequencing revealed that this virus is a relatively small myovirus with a 64847 bp long dsDNA genome, which is consistent with virion electron microscopy data. Bacteriophage Gekk3-15 is distinct in its nucleotide and encoded aa sequences from all other sequenced bacteriophage genomes, and may represent a new viral taxon at the genus or subfamily level.

## Introduction

Currently, aquaculture is the primary producer of nutritional fish in most developed countries. ^1^ The cultivation of hydrobionts in production ponds is associated with very high densities of animal populations, which far exceed the typical population densities in wild habitats. Such conditions make cultivated fish specifically vulnerable to bacterial infections, of which aeromonoses comprise a significant fraction. ^2^^,^^3^ The use of antibiotics to reduce economic losses associated with bacterial infections is restricted or prohibited in many countries; therefore, using phages as a tool for environmentally friendly biocontrol of the iсhtyopathogenic *Aeromonas* has attracted significant interest in scientific and business communities. Therefore, the search for new bacteriophage strains that are potentially suitable for use as phage therapy agents is important. Here, we present the isolation and sequencing of a novel *A. veronii* bacteriophage that represents a new group of bacteriophages that infect the genus *Aeromonas.*

## Methods

### Phage isolation and cultivation

The bacteriophage Gekk3-15 was isolated as part of a project to establish a panel of bacteriophages to target the collection of *A. hydrophila* and *A. veronii* strains that cause infections in aquaculture facilities in Russia. The phage was isolated from a water sample collected from the Norishka Stream in Mikhailovsky Park in Moscow, Russia, on September 14, 2021. The bacteriophage was isolated using the enrichment culture setup as follows: A non-sterilized water sample (0.5 l) was placed in a 2 l sterile plastic bottle and supplemented with 50 ml of sterile 10x LB medium (for 1× medium: 10 g Bacto Tryptone (Amresco, Am-J859-0.25), 5 g yeast extract (Amresco, J850-500G), 10 g NaCl (Amresco, J869-500G), distilled H _2_O up to 1 l). The culture was supplemented with 0.5 ml of an overnight liquid culture of *A. veronii* AV-3 strain and incubated at 28 °C in a thermostat without agitation. Following incubation, 1 mL of the enrichment culture was centrifuged at 12 000 rpm in a tabletop microcentrifuge, filter-sterilized using a 0.22 μm syringe membrane filter (Millex-GP, Millipore), and plated onto the lawn of *A. veronii* strain 4 using the conventional double-layer technique. ^4^

Phage plaques were formed after an overnight incubation at 28°C. The phage was purified using two consecutive single-plaque isolations. To obtain a high-titer lysate, five 90 mm Petri dishes were filled with solid LB medium (15 g of Bacto Agar (BD214030, BD Difco) per 1 l). Without drying the plates, they were overlaid with 5 ml of soft LB agar (6 g Bacto Agar (BD214030, BD Difco) per 1 l) per plate. Soft agar was inoculated with 300 μl of a 4h log-phase liquid culture of the host strain and approximately 1×10 ^5^ PFU of the phage per plate. The plates were incubated overnight at 28 °C. To extract the phage, the soft agar layer was gently destroyed with a spreader, transferred into 50 ml plastic centrifuge tubes, and layered with 10 ml of LB medium. Chloroform (Fisher Scientific, C298-4)(100 μL) was added to each tube, followed by vigorous vortexing for 1 min, and allowed to stand at room temperature for 2 h. After incubation, the agar fragments and bacterial cells were pelleted by centrifugation at 10 000 g for 5 min, and the supernatant was collected and centrifuged again under the same conditions. The phage was purified using a sucrose gradient as it described elsewhere. ^5^ The purified phage sample was used for DNA extraction and transmission electron microscopy (TEM), as previously described. ^6^

### DNA extraction, sequencing, assembly and annotation

To extract DNA, the phage stock with a titer of approximately 10 ^11^ PFU ml ^−1^ was treated with DNAse (Thermo Scientific, EN0521)(0,01 mg ml ^−1^) for 30 min at room temperature, and the phage particles were then collected by ultracentrifugation in an angle rotor at room temperature (Beckman 45Ti, 1 h, 75000 g). Genomic DNA was extracted from the precipitates with CTAB cetyltrimethylammonium bromide (CTAB) extraction as described previously. ^7^ DNA quality and quantity were assessed using agarose gel electrophoresis and a Qubit dsDNA HS fluorometer assay (Qubit, USA). Phage genomic DNA libraries were prepared and sequenced using an Ion Proton sequencer (Applied Biosystems, Foster City, CA, USA) with the standard chemistry according to the manufacturer’s instructions. The raw reads from the run were combined and filtered using the error correction tool Pollux ( https://github.com/emarinier/pollux). Contigs were assembled using Newbler version 3.0 (RRID:SCR_011916) (Roche Diagnostics, USA). A single contig with a phage genome of 64 847 b.p. with an average coverage of 200 bp was obtained.

Annotation was performed using Prokka (RRID:SCR_014732) ^8^ with subsequent manual curation. Potential open reading frames (ORFs) were detected using GeneMarkS ( https://genemark.bme.gatech.edu/genemarks.cgi)(RRID:SCR_011930) and subsequently analyzed using HMMER (RRID:SCR_005305), ^9^ HHPRED (RRID:SCR_010276) ^10^ (MPI Bioinformatics Toolkit), NCBI BLAST (RRID:SCR_004870), ^11^ and tRNAscan-SE (RRID:SCR_008637). ^12^

## Results

**Figure 1.**
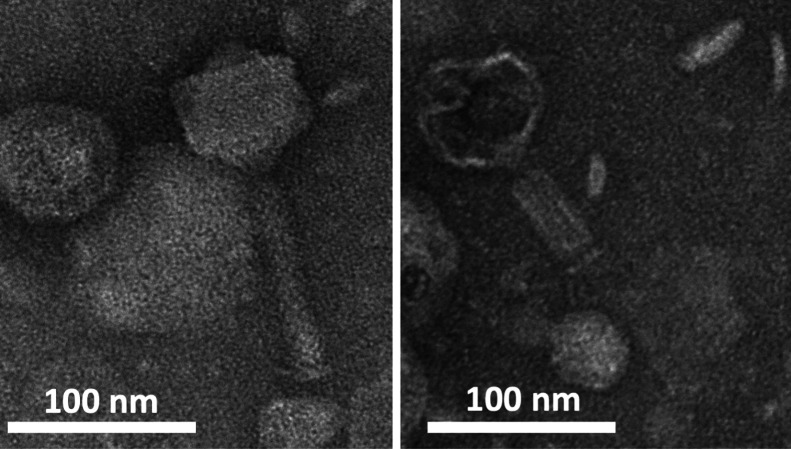
Morphology of the Gekk3-15 phage virions with extended (left) and contracted (right) tail.

The bacteriophage Gekk3-15 was found to be a relatively small myovirus with an isometric head ( Figure 1). The genome consisted of 64 847 b.p. and encoded 101 ORF, including putative virion structural proteins. A set of proteins for the contractile phage tail was detected, confirming the bacteriophage TEM examination data. The Gekk3-15 genome also contains genes for DNA metabolism enzymes, a cell lysis system, and two potential auxiliary metabolic genes (AMGs), encoding 3-oxoacyl-[acyl-carrier-protein] reductase FabG and glyoxylase/dioxygenase superfamily proteins. No tRNA genes were identified. Phage Gekk3-15 does not contain any markers potentially associated with a lysogenic lifestyle or virulence factors. A BLASTN (RRID:SCR_001598) search against the NCBI nucleotide collection did not produce any relevant hits for related bacteriophage genomes. However, the BLASTX (RRID:SCR_001653) search identified a number of distantly related viruses, among which the closest relative by the large terminase a.a. sequence was the *Pseudomonas phage* EPa61 (GenBank NC_048744), sharing 57% aa identity with the Gekk3-15 protein. The levels of a. a. identity between the structural proteins of bacteriophages Gekk3-15 and EPa61 varied between 38% and 63%. This degree of similarity indicates that taxonomically, these phages belong to different genera and, probably, different subfamilies within the Caudoviricetes kingdom. Therefore, we conclude that phage Gekk3-15 represents a novel genus and, probably, a novel bacteriophage taxon of a higher rank.

## Ethics and consent

Ethical approval and consent were not required.

## Data Availability

NCBI GenBank: Aeromonas phage Gekk3-15, complete genome. Accession number OR661252; https://www.ncbi.nlm.nih.gov/nuccore/OR661252.1. ^13^
