# Supplementary material for: Isolation and complete genome sequence of Aeromonas bacteriophage Gekk3-15
Source: F1000Res. 2024 Apr 24;13:380. [Version 1] doi: 10.12688/f1000research.144007.1 (PMC11372344; doi:10.12688/f1000research.144007.1)
Supplement: Peer review history

# Reviewer report: Reviewer response for version 1

**DOI:** 10.5256/f1000research.157743.r278896

**Published:** 2024-09-03

**Authors:** Reham M El Tarabili

**Affiliations:** 1 Bacteriology, Immunology and Mycology Department, Suez Canal University, Ismailia, Ismailia Governorate, Egypt

The authors declared, “Isolation and complete genome sequence of *Aeromonas* bacteriophage Gekk3-15”. Despite the importance of the study, the article lacks a good presentation. It has many grammar and language mistakes.

 The order of event writing should be the same either in the abstract, introduction, material, ……….so on.

 A title should be modified

 - Please write the scientific names of bacterial pathogens and genes in the correct form (italic form) all over the manuscript and the references section.

Are the datasets clearly presented in a usable and accessible format, and the assembly and annotation available in an appropriate subject-specific repository?

Yes

Are sufficient details of the sequencing and extraction, software used, and materials provided to allow replication by others?

Yes

Are the rationale for sequencing the genome and the species significance clearly described?

Yes

Are the protocols appropriate and is the work technically sound?

Yes

Reviewer Expertise:

microbiology ,immunology , mycology , molecular biology and food safety

I confirm that I have read this submission and believe that I have an appropriate level of expertise to confirm that it is of an acceptable scientific standard.

# Reviewer report: Reviewer response for version 1

**DOI:** 10.5256/f1000research.157743.r278893

**Published:** 2024-06-17

**Authors:** Ben Temperton, Liam Boot-Handford

**Affiliations:** 1 University of Exeter, Exeter, England, UK

**Editorial Note from the F1000Research Editorial Team: This peer-review report was originally published on 17th June 2024, and on 1st July 2024 the report was edited to include an additional paragraph provided by the reviewer relating to Reviewer 1's (Ruben Avendano-Herrera's) report for this article.**

**Review for: Isolation and complete genome sequence of *Aeromonas* bacteriophage Gekk3-15**

 This work details the method of isolation and sequencing of a novel bacteriophage that infects *Aeromonas veronii*, as well as translations of the genome. This is a useful finding as the isolated phage shares a low degree of similarity to previously sequenced phages, and the most closely related phage infects a host belonging to a different genus.

**Major comments:**

1. The framing of the study may be too broad for the content. The abstract discusses the use of phage in *Aeromonas* *hydrophila, *but this is not tested in the main text, and it is unclear if this particular phage would infect this host. We recommend either re-framing the abstract to specify *Aeromonas veronii* only, as this is the only isolate tested, or to test the phage infectivity on *A. hydrophila *also. This is especially important if the aim of the phage isolation is to be used in phage therapy.

**Minor comments:**

 Page 3, under Methods, 'Phage isolation and cultivation', paragraph 1: "at 28 deg. Celsius in a thermostat without agitation". "at 28 deg. Celsius in a thermostatic incubator without agitation" would be clearer.

 Same paragraph: please add how many minutes the enrichment culture was centrifuged for. The x g should be listed rather than rpm

 Same paragraph: it is unclear what *A. veronii* strain was used to isolate this phage, initially it is listed as AV-3 strain, and later as strain 4. Either these are different strains or one was mis-named in the manuscript, but regardless it would be pertinent to introduce any strains used and include information about the origin(s).

 Page 3, under Methods, 'Phage isolation and cultivation', paragraph 2: what method was used for single-plaque isolations? Please provide a reference.

 Same paragraph: should read 'as described elsewhere' not 'as it described'.

 Page 3, under Methods, 'DNA extraction, sequencing, assembly and annotation', paragraph 1: should read '0.01 mg ml ^-1^' not '0,01 mg ml ^-1^'

 Please use 'L' rather than 'l' for litres as this removes possible confusion between 'l', 'I', and 1.'L' is the approved symbol for litre ( https://www.nist.gov/pml/owm/si-units-volume).

 The authors attempt to classify their phage into an appropriate taxonomy based on amino acid identity of structural proteins. While this is not the gold standard as recommended by the ICTV (nucleotide similarity across the genome as specified by VIRIDIC), it is probably as close as is possible, given the paucity of suitably similar reference genomes. For instance we attempted to identify the closest phage genome in both INPHARED (Cook et al., 2021 ^1^) and PhageClouds (Rangel-Pineros *et al.*, 2021 ^2^) (at both 0.15 and 0.2 distance), which yielded no hits. Therefore, the cautious interpretation of the placement by the authors is appropriate and should be revisited when more closely related genomes are deposited.

 The reviewer Ruben Avendano-Herrera is incorrect in requesting that the authors use the term Myoviridae. This term was abandoned by the ICTV in their latest guidance which now requires the use of 'myovirus' to describe morphology, due to a lack of evidence for a shared common ancestor (Turner *et al.*, 2023[ref-3).

Are the datasets clearly presented in a usable and accessible format, and the assembly and annotation available in an appropriate subject-specific repository?

Yes

Are sufficient details of the sequencing and extraction, software used, and materials provided to allow replication by others?

Partly

Are the rationale for sequencing the genome and the species significance clearly described?

Yes

Are the protocols appropriate and is the work technically sound?

Yes

Reviewer Expertise:

phage biology; phage bioinformatics

We confirm that we have read this submission and believe that we have an appropriate level of expertise to confirm that it is of an acceptable scientific standard.

## References

[1] : INfrastructure for a PHAge REference Database: Identification of Large-Scale Biases in the Current Collection of Cultured Phage Genomes. *Phage (New Rochelle)* .2021;2(4) : 10.1089/phage.2021.0007 214-223 10.1089/phage.2021.0007 36159887 PMC9041510

[2] : From Trees to Clouds: PhageClouds for Fast Comparison of ∼640,000 Phage Genomic Sequences and Host-Centric Visualization Using Genomic Network Graphs. *Phage (New Rochelle)* .2021;2(4) : 10.1089/phage.2021.0008 194-203 10.1089/phage.2021.0008 36147515 PMC9041511

[3] : Abolishment of morphology-based taxa and change to binomial species names: 2022 taxonomy update of the ICTV bacterial viruses subcommittee. *Arch Virol* .2023;168(2) : 10.1007/s00705-022-05694-2 74 10.1007/s00705-022-05694-2 36683075 PMC9868039

# Reviewer report: Reviewer response for version 1

**DOI:** 10.5256/f1000research.157743.r283363

**Published:** 2024-06-10

**Authors:** Ruben Avendaño-Herrera

**Affiliations:** 1 Universidad Andres Bello, Santiago, Santiago Metropolitan Region, Chile

The manuscript is well written and requires the modifications indicated below. On the other hand, the title is too generic, considering that it only has activity against one species of *Aeromonas*, especially one isolate. The title needs to be more specific. It is suggested to modify it.Â The main issue is that the conclusion is too speculative, and further evidence is needed to confirm whether it corresponds to a new genus. Therefore, this situation should be verified and confirmed, or else it should be concluded that the bacteriophage has activity against an *Aeromonas* isolate. There are results that are not provided and are indicated at the end of this evaluation.

 Abstract

 Change to 64,847 bp

 Amino acids (aa)

 Keywords

 Italicize *Aeromonas veronii*

 Introduction Reference No. 1 could be replaced by the FAO report or another article more relevant to the statement.

 "Aeromonoses" seems to be a term not globally used. Replace it with " *Aeromonas* infections" or " *Aeromonas* diseases."

 Bacteriophage isolates

 Â How many are the isolates from the collection of the two *Aeromonas* species?

 LB, the full name of the medium should be provided before the abbreviation.

 How was it identified that AV-3 corresponds to the species *A. veronii*?

 This should be assured, as the taxonomy of this group is not easy and requires molecular analysis. Make this clarification for all *Aeromonas* used in this study.

 "Strain" or "isolates," I think the latter. Review throughout the article.

 It is not necessary to give the LB recipe twice; just indicate the agar percentage in the last case.

 What concentration of DNA was sent for sequencing? This data is very important.

 Specify what "bp" is and correct the abbreviation, removing the periods.

 Results

 Indicate that it belongs to the family *Myoviridae* instead of "myovirus" in italics.

 The last lines where the conclusion is drawn, I believe it is too speculative to state that it corresponds to a new genus and further tests are required to confirm it.

 The article should modify the conclusion and reduce the enthusiasm, unless there are other tests that prove it. I suggest modifying it. If it was tested with a collection of Aeromonas, I would like to know if the activity is broad-spectrum or if it's only an isolate that was analyzed. This situation is very important.

 Additionally, was any physiological characterization of the virus conducted, considering that the introduction suggests they are an alternative for use in fish aquaculture?

Are the datasets clearly presented in a usable and accessible format, and the assembly and annotation available in an appropriate subject-specific repository?

Partly

Are sufficient details of the sequencing and extraction, software used, and materials provided to allow replication by others?

Partly

Are the rationale for sequencing the genome and the species significance clearly described?

Yes

Are the protocols appropriate and is the work technically sound?

Yes

Reviewer Expertise:

Some of my main research topics include pathology in aquaculture; diseases of aquatic organisms; biotechnological tools applied to aquaculture; standardization of procedures to control and validate the appropriate use of antibiotics; and the development of vaccines against aquatic pathogens.

I confirm that I have read this submission and believe that I have an appropriate level of expertise to confirm that it is of an acceptable scientific standard, however I have significant reservations, as outlined above.
